# Knowledge, Attitudes, and Practices Regarding Food Habits Among Pregnant Tribal Women in Santhal Pargana, Jharkhand: A Cross-Sectional Pilot Survey

**DOI:** 10.7759/cureus.90859

**Published:** 2025-08-24

**Authors:** Anita Kumari, Varnibha Pant, Sanjeet Kumar Singh, Amita Kumari, Kumar A Prasad, Pradosh Kumar Sarangi, Himel Mondal

**Affiliations:** 1 Physiology, All India Institute of Medical Sciences, Deoghar, IND; 2 Medicine, All India Institute of Medical Sciences, Deoghar, IND; 3 Pathology, All India Institute of Medical Sciences, Deoghar, IND; 4 General Medicine, Community Health Center, Deoghar, IND; 5 Radiodiagnosis, All India Institute of Medical Sciences, Deoghar, IND

**Keywords:** anemia, health knowledge, malnutrition, micronutrients, pregnancy, pregnant women, tribal woman

## Abstract

Introduction: Maternal malnutrition is common in developing countries. They face deficiencies in macronutrients and micronutrients. This study aimed to evaluate the knowledge, attitudes, and practices related to nutrition among tribal pregnant women in a developing district in Jharkhand, India.

Methods: A cross-sectional survey was conducted with a purposive sample of 139 pregnant tribal women attending a tertiary healthcare center in Deoghar, Jharkhand, India. Data were collected using a structured questionnaire. An expert surveyor with local language proficiency collected the data. The questionnaire collected data on sociodemographic characteristics, obstetric history, and nutrition-related knowledge, attitudes, and practices. There were seven questions for each domain, totaling 21 questions. For each domain (having seven questions with a maximum score of seven), the total score was divided by seven to get an average score.

Results: Among the 139 participants, 107 (76.97%) were aged between 15 and 24 years. Most women (91, 45.46%) had education up to the secondary level, while 124 (89.2%) were homemakers. Primigravida women accounted for 61 (43.88%) participants. Anemia was prevalent (average hemoglobin 9.51±0.48 gm/dL) among all participants. The knowledge score was 1.75±0.57, attitude 1.04±0.24, and practice 1.36±0.02 (ANOVA F = 125.6, P < 0.0001). A multiple linear regression model indicated that knowledge and attitude collectively predicted practice (R = 0.45, P < 0.0001), but only knowledge made a significant independent contribution (t = 4.4, P < 0.0001). Similarly, age, education, income, and gravida significantly predicted knowledge levels (R = 0.828, P < 0.0001), with education being the only significant independent predictor (t = 15.73, P < 0.0001).

Conclusion: Despite a high prevalence of anemia, participants exhibited limited knowledge, attitude, and practice of food habits among pregnant tribal women. The level of education plays a crucial role in shaping knowledge, attitudes, and practices. It emphasizes the role of awareness programs in improving maternal health.

## Introduction

Malnutrition among women of reproductive age arises from an imbalance between the increased nutritional demands of pregnancy and lactation and inadequate dietary intake [1]. Adequate nutrition during pregnancy is vital for the health of both mother and fetus, influencing pregnancy outcomes, childbirth, and breastfeeding [2]. Poor dietary intake during pregnancy can lead to irreversible effects, including maternal malnutrition, anemia, low birth weight, and preterm birth. Findings from the fifth National Family Health Survey (NFHS) reveal that approximately 61% of women of reproductive age in aspirational districts in India suffer from anemia [3].

Food restrictions, misconceptions, frequent illnesses, and inadequate care significantly contribute to malnutrition in pregnant and breastfeeding women. These factors, combined with insufficient dietary intake and unequal sharing of household food resources, exacerbate the issue [4]. Research examining the nutritional understanding and behaviors of expectant mothers reveals that a wide array of beliefs rooted in knowledge systems significantly shape dietary habits [5,6].

In some states, more than 50% of tribal people suffer from anemia. A study reported that 95.3% of tribal women suffer from anemia [7]. This may be further complicated during pregnancy due to higher nutritional demand [8]. Hence, it is necessary to assess the knowledge, attitudes, and practices regarding food consumption among tribal women and to identify the factors associated with a higher risk of developing anemia. Hence, this pilot study aimed to assess the knowledge, attitude, and practices of food habits during pregnancy in tribal women.

## Materials and methods

This cross-sectional pilot study was conducted among pregnant tribal women in Deoghar, Jharkhand, over two months (November 2023 to December 2023). The study was carried out in a tertiary healthcare center catering to a tribal population area. The study received ethical approval from the Institutional Ethical Committee of the All India Institute of Medical Sciences, Deoghar, in September 2023 (reference number: 2023-224-EMP-03, approval date 06/09/2023). Written informed consent was obtained from all participants before data collection. Data confidentiality was maintained by anonymizing all collected information.

This study was designed as a pilot survey aimed at exploring preliminary trends and feasibility, and therefore did not require formal sample size calculation. The focus was on gaining initial insights, which will inform the design of a larger, more definitive study in the future.

A total of 139 pregnant tribal women who met the predefined inclusion criteria were selected for the study using a purposive sampling technique, which allowed the researchers to specifically target individuals most relevant to the research objectives. The participants included women across all three trimesters of pregnancy who were attending antenatal care services for the first time during their current pregnancy. This approach ensured that the data captured early maternal health-seeking behavior and perceptions among tribal women. To maintain the study’s focus and reduce confounding variables, women presenting with acute illnesses, those identified as high-risk pregnancies (based on clinical assessment), and those who declined to participate were excluded from the sample.

A questionnaire was developed based on a literature review [1,3,5] and expert consultation to ensure content validity. It was designed to assess sociodemographic characteristics, obstetric history, and nutrition-related knowledge, attitudes, and practices. In this study, knowledge refers to the awareness of recommended foods, supplements, and dietary needs during pregnancy; attitude denotes the beliefs and perceptions of pregnant tribal women toward healthy eating and food taboos; and practices represent the actual dietary behaviors such as meal frequency, dietary diversity, iron-folic acid consumption, and avoidance of food restrictions. The questionnaire comprised 21 items, with seven questions in each domain. Each question carried a maximum of one mark, and an incorrect answer was scored as 0. The questions and their measurements are available in Appendix A.

Descriptive statistics were used to present data in numbers and percentages. For scoring the response, we scored “one” for a correct answer and “0” for a wrong answer. ANOVA was conducted to compare the mean scores across knowledge, attitude, and practice domains in order to determine how knowledge levels may shape attitudes and translate into actual practices. A regression analysis was conducted to evaluate the predictive relationships between age, education level, parity, income, and knowledge, attitudes, and practices scores. Additionally, the ability of knowledge scores to predict attitudes and practices scores was assessed. A p-value < 0.05 indicated a statistically significant result. The statistical analysis was conducted using GraphPad Prism 9.5.0 (GraphPad Software, La Jolla, CA, USA).

## Results

The demographics of the participants are shown in Table 1. The study population consisted of young women, with most engaged in household responsibilities. Education levels varied, but the majority had received secondary schooling, while none had attained higher education. The predominant occupations among husbands were farming and labor work. Household incomes were generally above subsistence levels, though a small fraction earned lower wages. A significant portion of the participants had previous pregnancies. Notably, all participants exhibited anemia, highlighting a critical nutritional concern.

**Table 1:** The sociodemographic and obstetric characteristics of pregnant mothers

Variable	Categories	Frequency	Percentage
Age (years)	15-24	107	76.97
	25-34	31	22.30
	35-44	1	0.72
Occupation	Homemaker	124	89.20
	Farmer	13	9.35
	Labour worker	2	1.43
Education	Did not attend school	15	10.79
	Primary school	33	23.74
	Secondary school	91	65.46
Husband’s occupation	Farmer	71	51.07
	Labor worker	34	24.46
	Business	25	17.98
	Others	9	6.47
Monthly income (Rupees)	1000-5000	3	2.14
	>5000	136	97.84
Gravida	Primiparous	61	43.88
	Multiparous	78	56.11
Hemoglobin	<11 gm/dL	139	100

**Figure 1:**
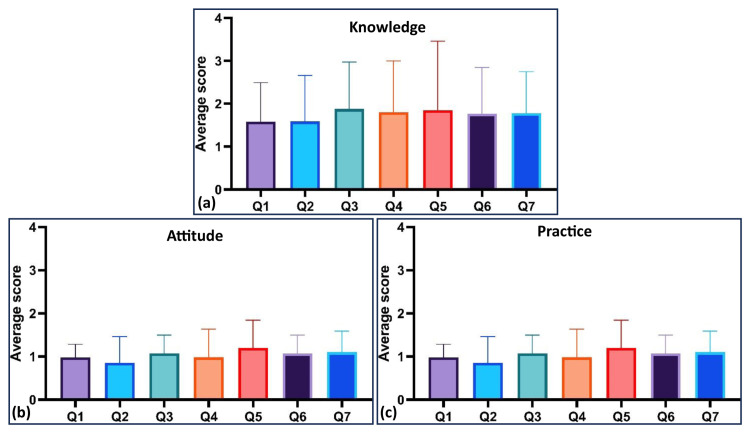
Question-wise scores for (a) knowledge, (b) attitudes, and (c) practices The average score indicates the mean score of each question in three domains (knowledge, attitude, and practice). Q1-Q7 indicates the number of questions from one to seven. Q: question

The knowledge score was 1.75±0.57, the attitudes score was 1.04±0.24, and the practices score was 1.36±0.02 (ANOVA F = 125.6, P < 0.0001). There was a significant difference among knowledge, attitudes, and practices scores (ANOVA F = 125.6, P < 0.0001). Post-hoc analysis confirmed significant differences between all pairs: knowledge versus attitudes had a mean difference of 0.71 (95% CI: 0.61 - 0.82, P <0.0001), knowledge versus practices had a mean difference of 0.39 (95% CI: 0.28 - 0.49, P <0.0001), and attitudes versus practices had a mean difference of -0.32 (95% CI: -0.43 to -0.22, P <0.0001). This indicates that women’s knowledge, attitudes, and actual food practices were not the same. Knowledge scores were higher than both attitudes and practices, and attitudes were also higher than practices. This means that even if women know what is good and feel positive about it, it does not always translate fully into daily habits. Figure 1 shows the question-wise scores in knowledge, attitudes, and practices.

Knowledge scores did not significantly differ among individual questions (ANOVA F = 1.46, P = 0.19) (Figure 1). However, attitude scores showed significant variation (ANOVA F = 6.189, P < 0.0001), with six significant differences among 21 question pairs. Practice scores also demonstrated significant variation (ANOVA F = 3.02, P = 0.006), with four significant differences among 21 question pairs.

**Figure 2:**
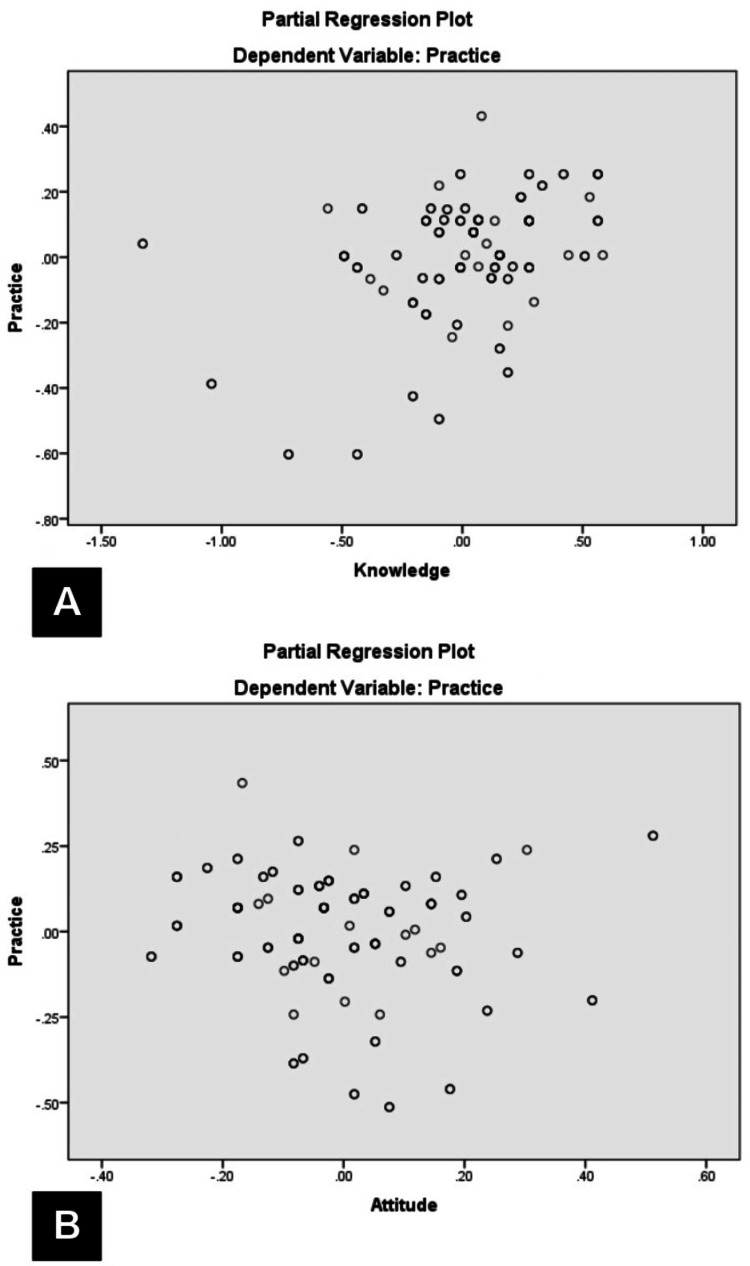
Partial regression plot between knowledge and practices (A) and attitudes and practices (B)

A multiple linear regression model indicated that knowledge and attitudes collectively predicted practices (R = 0.45, P < 0.0001), but only knowledge made a significant independent contribution (t = 4.4, P < 0.0001). A partial regression plot is shown in Figure 2.

**Figure 3:**
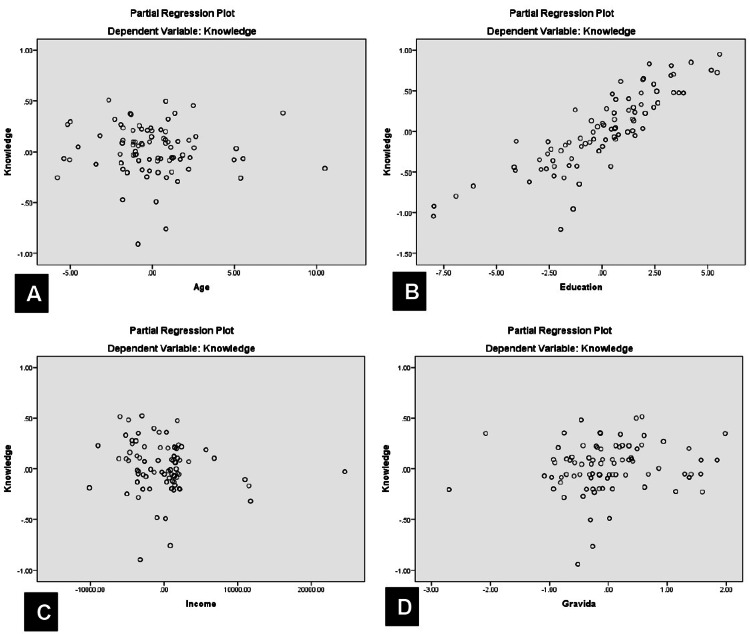
Partial regression plot of knowledge versus age (A), education (B), income (C), and (D) gravida

Similarly, age, education, income, and gravida significantly predicted knowledge levels (R = 0.828, P < 0.0001), with education being the only significant independent predictor (t = 15.73, P < 0.0001). The partial regression plot is shown in Figure 3. 

**Figure 4:**
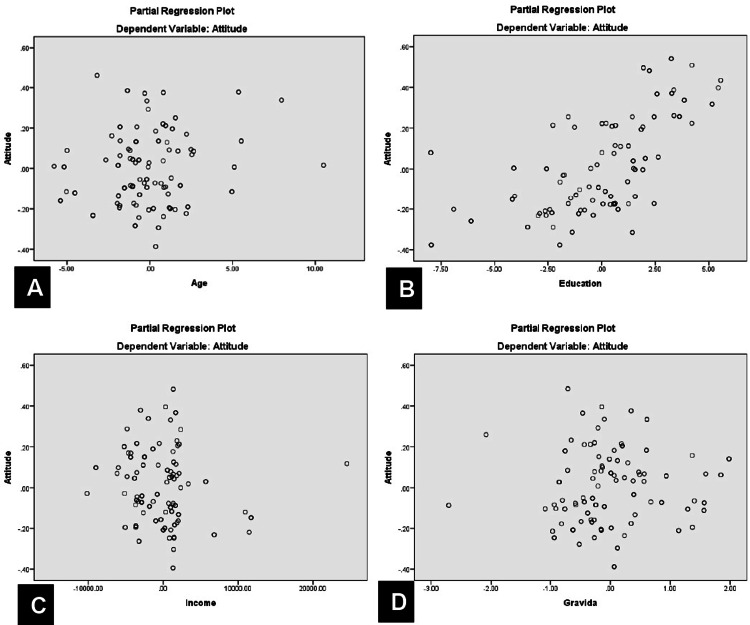
Partial regression plot of attitude versus age (A), education (B), income (C), and (D) gravida

**Figure 5:**
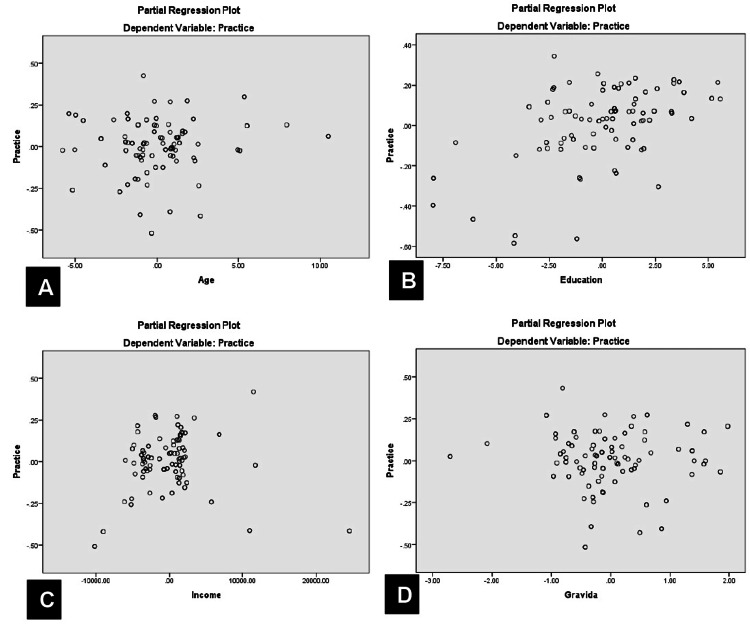
Partial regression plot of practice versus age (A), education (B), income (C), and (D) gravida

Likewise, these demographic factors also predicted attitudes (R = 0.627, P < 0.0001) (Figure 4) and practices (R = 0.563, P < 0.0001) (Figure 5), but education remained the sole significant independent predictor for both (t = 8.87, P < 0.0001 for attitude; t = 7.38, P < 0.0001 for practice).

## Discussion

This study assessed the knowledge, attitudes, and practices regarding nutrition during pregnancy among tribal pregnant women and identified key sociodemographic determinants. The findings revealed that a significant proportion of participants were young, primarily homemakers, and had limited educational attainment beyond secondary school. While the majority had some awareness of nutrition, a considerable percentage lacked sufficient knowledge. Attitudes and practices scores also showed significant variations, with education emerging as the strongest predictor for all three components. The study further highlighted the widespread prevalence of anemia among pregnant women despite iron supplementation. Hence, there is a crucial role of maternal education in shaping nutritional awareness and practices [9].

The proportion of women lacking knowledge in this study was lower than that reported in rural Punjab [10] but higher than figures observed in Lagos [11], suggesting that regional differences significantly impact awareness levels. However, these studies did not report the knowledge levels in tribal women. Hence, our study suggests that tribal women staying in rural areas may have lower knowledge when compared to non-tribal women. Similarly, the percentage of participants with unfavorable attitudes toward nutrition was lower than that seen in Addis Ababa [12] but higher than among Syrian refugees [13]. These variations may stem from differences in geographical location, population characteristics, educational exposure, cultural beliefs, access to healthcare, and public health initiatives. Regarding nutritional practices, a substantial proportion of participants exhibited inadequate behaviors, though this was less pronounced compared to findings among Syrian refugees [12]. Such discrepancies highlight the influence of sociodemographic factors, food availability, and public health interventions on maternal dietary habits.

All the women had anemia in our study. This is higher than a study by Dwivedi et al. conducted in Rajasthan, which reported that about 86% of tribal pregnant women suffer from anemia [14]. In our study, despite reported consumption of iron-rich foods and supplements, all had anemia. Hence, their response to their practice may not truly capture their practice, and they might be influenced by social desirability bias in response [15]. In addition, this may be suggestive of potential issues such as poor dietary absorption, underlying health conditions, or inconsistencies in supplement adherence [16]. Education appears to play a crucial role in dietary choices, healthcare-seeking behavior, and adherence to nutritional recommendations. Women with lower education levels may have limited access to reliable health information, potentially contributing to poor dietary diversity and persistent micronutrient deficiencies [17].

The findings have important public health implications, particularly for designing culturally sensitive nutrition interventions. Understanding the sociocultural and economic barriers influencing dietary behaviors can inform targeted educational programs to improve maternal nutrition among tribal populations [18]. Addressing gaps in knowledge and challenging traditional food taboos through community-based initiatives can promote healthier dietary practices in this area and similar underserved regions [19]. Strengthening maternal education programs can have a profound impact on improving nutritional status and pregnancy outcomes, ultimately contributing to long-term public health improvements [20].

The observed patterns of nutritional knowledge, attitudes, and practices are not merely reflections of personal awareness but are deeply embedded within the socio-ecological fabric shaped by marginalization [21], health service access [22], and intergenerational educational inequalities [23]. The universal prevalence of anemia despite iron supplementation underscores systemic gaps in nutrition program implementation, bioavailability of nutrients, and possibly, parasitic or gastrointestinal comorbidities common in resource-limited tribal areas [24]. Thus, any effective intervention must adopt a multisectoral approach that combines health education with structural supports such as food security, clean water, and accessible healthcare, tailored to the specific realities of tribal communities.

The study has several limitations. As a cross-sectional study, it captures data at a single point in time, limiting the ability to establish causality. Selection bias may also be present, as participation was voluntary and may not fully represent the entire pregnant tribal population. Additionally, reliance on self-reported data introduces the potential for recall bias or socially desirable responses. Seasonal variations in food availability and dietary patterns were not accounted for, which could influence the findings.

## Conclusions

This study highlights a substantial gap in knowledge, attitudes, and practices related to maternal nutrition among pregnant tribal women, despite a moderate level of formal education in many participants. Educational attainment emerged as a critical determinant, strongly influencing attitudes and behaviors. However, the widespread prevalence of anemia, despite reported consumption of iron-rich foods and supplements, points to deeper, multifactorial challenges beyond knowledge alone. The findings underscore the urgent need for integrated, culturally tailored interventions that go beyond informational campaigns to address structural issues affecting maternal nutrition.

## Appendices

Appendix A 

**Table 2:** Questions and its measurement The original questionnaire was in Hindi. The response options were different for different questions. The score of each domain was normalized to compare among the three domains. Each question had a score of a maximum of one and a minimum of 0. Hence, for each domain, the maximum score was seven and the minimum was 0. The average score in each domain was calculated by dividing the score across seven questions by seven.

Domain	Question	Measures
Knowledge	What do you think are the causes of malnutrition?	Understanding of the etiology of malnutrition — both dietary and non-dietary factors
	What supplements do you think pregnant women need during pregnancy?	Knowledge of recommended nutritional supplementation during pregnancy (e.g., iron, folic acid)
	How to identify anemia?	Awareness of clinical signs, symptoms, and diagnostic criteria of anemia
	What are the health risks when a pregnant woman is deficient in iron in her diet?	Understanding of the consequences of iron deficiency during pregnancy for mother and fetus
	What do you think are the measures to prevent anemia?	Knowledge of preventive strategies for anemia (dietary, supplementation, deworming, etc.)
	What are iron-rich foods?	Identification of common dietary sources of iron
	Which habits help maintain a healthy pregnancy weight?	Understanding of lifestyle and dietary behaviors that support healthy gestational weight
Attitude	How good do you think it is to eat more food during pregnancy?	Attitude towards increased nutritional intake during pregnancy
	How do you think eating more carbs is better during pregnancy?	Attitude towards carbohydrate consumption during pregnancy
	How good do you think it is to eat more protein or beans during pregnancy?	Attitude towards protein-rich food intake during pregnancy
	In your opinion, how good is it to consume more milk and milk products during pregnancy?	Attitude towards dairy product consumption during pregnancy
	How good is cooking from non-vegetarian food?	Attitude towards non-vegetarian food consumption
	How much do you like the taste of meat and other iron-rich foods or meals?	Personal preference and attitude towards iron-rich food taste
	How much do you like the taste of milk and milk products?	Personal preference and attitude towards dairy product taste
Practice	How often do you consume iron-rich foods during pregnancy?	Frequency of consumption of iron-rich foods during pregnancy
	How often do you take supplements during pregnancy?	Frequency of supplement intake during pregnancy
	How often do you consult a healthcare provider during pregnancy for nutritional advice?	Frequency of seeking professional advice regarding nutrition during pregnancy
	How often do you track your weight gain during pregnancy?	Frequency of weight monitoring during pregnancy
	How often do you engage in physical activity during pregnancy?	Frequency of physical activity during pregnancy
	How frequently do you consume fruits and vegetables during pregnancy?	Frequency of fruit and vegetable intake during pregnancy
	How often do you eat non-vegetarian foods during pregnancy?	Frequency of non-vegetarian food consumption during pregnancy
